# Non-Orthogonal Multiple Access for Unicast and Multicast D2D: Channel Assignment, Power Allocation and Energy Efficiency

**DOI:** 10.3390/s21103436

**Published:** 2021-05-14

**Authors:** Mariem Hmila, Manuel Fernández-Veiga, Miguel Rodríguez-Pérez, Sergio Herrería-Alonso

**Affiliations:** atlanTTic Laboratory, Faculty of Telecommunications Engineering, Universidade de Vigo, 36310 Vigo, Spain; mveiga@det.uvigo.es (M.F.-V.); miguel@det.uvigo.gal (M.R.-P.); sha@det.uvigo.es (S.H.-A.)

**Keywords:** multicast device-to-device communication, 5G and beyond, non-orthogonal communications

## Abstract

Non-orthogonal multiple access (NOMA) techniques have emerged in the past years as a solution to approximate the throughput performance of wireless communications systems to their theoretical capacity region. We consider in this paper an optimization-based model for multicast device-to-device (MD2D) communications where the channels are not orthogonal and may be (partially or fully) shared among the transmitters in each cluster. This setting leads naturally to the introduction of NOMA transmitters and receivers who use successive interference cancellation (SIC) to separate the superposed signals. To analyze the role of NOMA in MD2D, its performance impact, potential performance gains and possible shortcomings, we formulate a model that includes SIC operations in the decoders, so that higher rates can be attained when several sources transmit on the same channel(s). We also investigate the energy efficiency of the network (global and max-min) through a dynamic power control algorithm and present a centralized and a semi-distributed solution to these optimization problems. Through numerical simulations, we show that NOMA is able to improve both the sum-rate and the max-min rate of a MD2D network even from a small degree of resource sharing. Furthermore, these gains also improve the global energy efficiency on the network, but not always the max-min energy efficiency of the devices.

## 1. Introduction

5G and beyond networks are poised to achieve spectrum efficiency increases from five to fifteen times compared to current 4G technology and densities around a million devices per square kilometer [1]. Multicast Device-to-Device (MD2D) communication is a conceptually simple technique that allows users in close proximity to communicate directly without the intervention of a third party, such as a base station (BS) or an access point. These short-range communications incur lower latencies and require less transmission power or, conversely, achieve higher transmission rates for the same power, thus increasing energy efficiency. As a result, both the energy efficiency and the system capacity are improved, and MD2D can contribute significantly to meet the requirements of vast 5G infrastructures.

However, in underlay MD2D communications, simultaneous transmissions over the same resource blocks (RBs) increase interference and may limit the network performance in both metrics, sum-rate and energy efficiency. An engineered allocation of resources (i.e., frequency channels and power levels) is thus necessary to mitigate interference and maximize network performance, ideally in a distributed manner, so as to also minimize the overhead and the computation load at any central site. Since the spectrum bands in MD2D are heavily reused by multiple transmitters, including the normal cellular users (CUs), the channel between these and the receivers in different clusters/groups can be modeled as a multi-user broadcast channel (MU-BC) using orthogonal multiple access (OMA) by the transmitters. Interference management in this setting has been extensively investigated both through separate or joint channel allocation and power control techniques, i.e., treating the interference at the receivers as noise (e.g., [2,3,4,5]). In contrast, with Non-Orthogonal Multiple Access (NOMA) [6,7], receivers are able to separate superposed signals distinguishing them in the power domain, by forcing some users to use Successive Interference Cancellation (SIC). By SIC, a user fully and sequentially decodes the signals intended for other weaker users, subtracts these from the received signal, and finally recovers its own signal with much lower noise. Thus, NOMA offers high prospects to suppress interference at the receivers, and has been thoroughly studied in the 5G design stage (and since much earlier in the information theory literature [8]) with remarkable success for achieving high efficiency [9,10,11,12,13,14]. Therefore, NOMA appears to be a promising solution to the problems of high spectral and energy efficiencies for 5G and beyond, given its provable benefits over OMA [15,16].

In this work, we address the problem of joint power allocation and channel assignment in underlay MD2D communications when the receivers can exploit NOMA, a channel/RB can be used by multiple MD2D groups and a MD2D group needs to simultaneously transmit over several channels to meet its rate constraint. Although the use of NOMA and SIC receivers has already been analyzed in several works [17,18] for unicast D2D communications, this paper considers the performance evaluation of NOMA for MD2D, so it generalizes previous assumptions. We formulate the optimization problem of energy efficiency (EE) under throughput constraints either for network EE or for max-min fair (MMF) EE maximization, since aggregate network performance does not capture adequately the notion of individual fairness. To that end, we extend our prior mathematical framework in [4,19] and explicitly model the existence of SIC receivers in the system. In addition, the degree of resource sharing can be adapted by design through two simple parameters: the maximum number of MD2D groups per RB (the reuse degree) and the maximum number of RBs assigned to a MD2D group (the split factor). This resource allocation problem under NOMA and SIC is solved with a two-stage approach in a semi-distributed form, much as in [4], by a combination of mathematical optimization (fractional programming) and game theory. Our results quantify the improvements gains associated to NOMA and SIC in MD2D communications, showing that NOMA is able to attain higher sum-rate, global energy efficiency and max-min rate (all simultaneously) than orthogonal transmission modes, even though it does not systematically achieve better max-min energy efficiency. Moreover, the benefits of NOMA start to appear only when there is enough contention for the shared channels and are clearer if the degrees of freedom in using the shared channels are larger.

The rest of the paper is organized as follows. Section 2 discusses the relevant literature in the context of Device-to-Device (D2D) pair/groups, MD2D and NOMA. Section 3 illustrates the system model. Then, optimal power allocation is detailed in Section 4, followed by the description of both the centralized and the distributed channel assignment solutions. Numerical results for the proposed schemes are given in Section 5. Section 6 contains some discussion and considerations on the role of NOMA in MD2D systems. Finally, concluding remarks are presented in Section 7.

## 2. Related Work

Both NOMA techniques and D2D communications have the potential to significantly improve energy and spectral efficiency (EE and SE, respectively) in 5G networks and beyond without demanding any modification to the deployed infrastructure [20]. However, integrating D2D technology into 5G comes with a set of technical hurdles, mainly, the co-channel interference between D2D communications and between D2D and other CUs served by the BS [21]. In a scenario where both D2D technology and NOMA are in use, the co-channel interference caused by D2D transmissions adds new challenges to the power allocation of CU transmissions.

Joint power control and channel allocation is investigated in [17] for maximizing the sum-rate of D2D pairs in a unicast transmission mode, where D2D nodes underlay a coexistent NOMA-based cellular network. A dual-based iterative decomposition approach of the optimization problem is developed to simplify the solution and determine the optimal transmission power for a set of CUs over the channels, and next the power of D2D transmitters and their allocated channels are selected. The combination of NOMA for the cellular users and D2D has also been explored in the mobile edge context (e.g., [22]) as a strategy to offload computing tasks from the edge servers. The goal is to minimize the weighted sum of users’ energy consumption and the computation delay. As another example, Wang et al. [23] incorporated NOMA-based D2D in the design of an advanced H-CRAN for 5G. Other works extend the setting to the case wherein both CUs and D2D receivers can exploit NOMA to improve system performance metrics, as in [24] (maximization of the sum rate) and [25] (minimization of the sum-power in the network). However, Zhao et al. [24] used a fixed power allocation, hence it does not realize all the potential gains, while Yoon et al. [25] relied on heuristics to solve the channel assignment problem.An efficient solution for power control and channel allocation is presented in [26,27], limited to the case in which a set of D2D pairs (D2D groups) is assigned to only one resource block. An alternative is to separate the regimes based on the aggregate interference level in the network, and use D2D with SIC only when the interference is low. This is explored in [28], but the model turns out to be an impractical combinatorial problem hard to solve that needs substantial simplifications. A mixed communication system that restricts NOMA to the D2D pairs and continues to use OMA for the transmissions between CUs and the BS is analyzed in [29] and solved used matching theory. Multicast D2D groups with NOMA are the focus of the works in [18,30], but they only consider groups with two receivers in order to simplify the analysis of the rates.

Compared with the state-of-the-art, the work presented in this paper introduces the following contributions:

1. The investigated system models in the literature assume a single/group of D2D pairs can share CUs resource blocks. In this unicast communication model, a transmitter sends data to a single receiver. However, in our system model, devices with a common interest form a group, where a single transmitter multicasts data to a set of receivers (the MD2D group). Note that the MD2D mode also includes the particular case of unicast D2D communications (when the groups just include one receiver).
2. In multicast communications, group data rate is determined according to the receiver with the poorest channel quality (CQ). Therefore, we assume that the receivers in a MD2D group as well as the CUs are able to apply SIC to the stronger interference signal. This would reduce the received interference to a minimum value leading to enhancements in MD2D communication quality.
3. We model the resource allocation sub-problem in underlay MD2D using matching theory and overlapping coalition formation game to minimize harmful mutual interference as the means to maximize energy efficiency for both the system as a whole and for individual users, metrics not considered in other related works.
4. The resource allocation approach involves two design parameters (the reuse degree and the split factor) which allow considering all the possible RB sharing variants when analyzing the system behavior.
5. The power control sub-problem is optimally solved using fractional programming. In this work, we assume that a central entity is in charge of assigning transmission power to each transmitter in the network.
6. We evaluate the performance of NOMA-based systems under a broad range of resource sharing scenarios. As performance metrics, we analyze both EE and transmission rate for the whole system (global EE and sum-rate). A potential drawback of this approach is that global performance measures do not properly capture the service fairness among users. Thus, we also formulate and analyze the MMF EE and the rate for individual users.

As stated, the sharing of resources includes the possibility that a user transmits over multiple channels and that a channel may be accessed by several transmitters. Assume that *r*, the *reuse degree*, represents the maximum number of transmitters per RB, and that *s*, the *split factor*, is the maximum number of allowed channels that a transmitter can use to increase its throughput. Both *r* and *s* are system parameters, which variations yield four distinguishable resource sharing scenarios:

**Scenario 1** 
(r=s=1): Each CU shares its communication channel with a single D2D pair/MD2D group. Similarly, each D2D pair/MD2D group uses only one channel.
**Scenario 2** 
(s>1, r=1): A D2D pair/MD2D group takes advantage of multiple cellular channels and distributes its message over them. Here, a D2D pair/MD2D group may disperse its transmission power budget or rate among the occupied channels. However, a channel cannot support more than one D2D pair/MD2D group.
**Scenario 3** 
(s=1, r>1): Each cellular communication channel can support up to *r* D2D pairs/MD2D groups, which are allowed to use one cellular channel, at most. Compared to the previous scenarios, having *r* devices using the same channel leads to mutual interference accumulation over the CU and each D2D pair/MD2D group per channel.
**Scenario 4** 
(s>1, r>1): Each D2D pair/MD2D group uses up to *s* different cellular channels. Moreover, each channel can support *r* D2D pairs/MD2D groups. As in Scenario 3, the accumulated mutual interference among the channel’s devices may negatively affect both type of communications and limit the benefits of D2D pairs/MD2D groups in term of spectral and energy efficiency.

For clarity purpose, we give each scenario a descriptive name: Scenario 1 (Dedicated CUs), Scenario 2 (Distributed Groups), Scenario 3 (Shared CUs) and Scenario 4 (General Case). We refer the reader to Table 1 for a summary of the related work explored in this Section using the proposed taxonomy.

## 3. Problem Formulation

### 3.1. System Model

**Figure 1:**
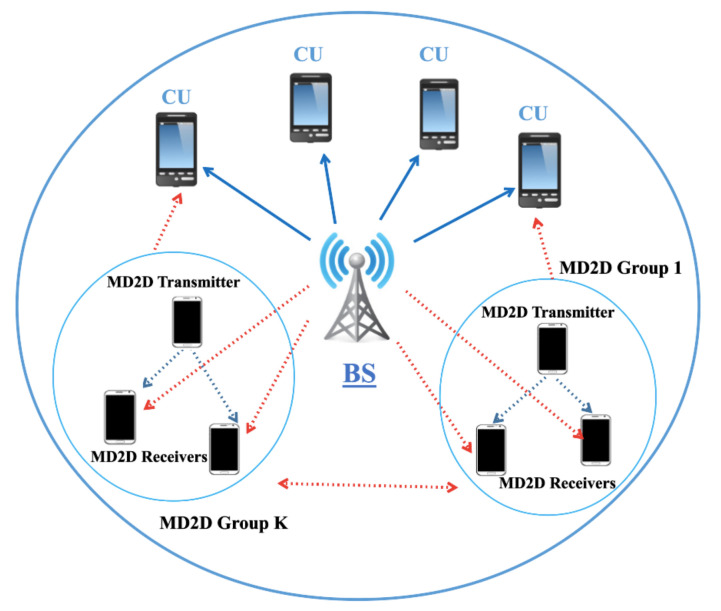
System model.

We consider a single-cell MD2D communications scenario with K≥2 MD2D groups of users and M≥2 CUs (see Figure 1). The BS communicates with the associated CUs over *M* orthogonal downlink channels. We identify each channel with an active CU in the downlink, thus the set of channels/CUs is denoted as $C=\{C_{1},\cdots ,C_{M}\}$. The set of receivers in group *k* is denoted by $D_{k}$, so $D=\{D_{1},\cdots ,D_{K}\}$ is the set of *K* MD2D groups. Any MD2D group, to increase its throughput, can simultaneously transmit over multiple channels up to a maximum of *s* channels, where *s* is the *split factor*; additionally, multiple MD2D groups can share the access to the same channel, up to a maximum of *r* groups per channel, where *r* is the *reuse degree* [4].

In this setting, the receivers in a given MD2D group have to cope with two kinds of interference:

- The CU interference is caused by the BS transmissions to the CU(s) over the channel(s) used by the MD2D group at the same time.
- The inter-groups interference is caused by the transmitters on those MD2D groups that are reusing the channel(s) the MD2D group is also accessing.

**Figure 2:**
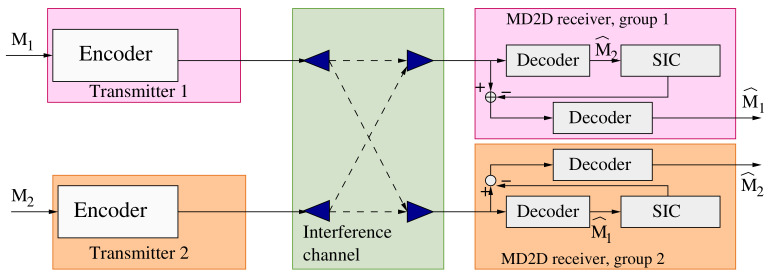
Communications architecture for the NOMA-SIC MD2D network.

Interference cancellation of superposed signals in the same time–frequency resource is possible through SIC, where at least one user is forced to fully decode the messages of the other co-channel users, subtract them from the received signal, and decode its own message afterwards. In information theory, receiver-side SIC and superposition coding (NOMA) are known to achieve the capacity region of the Single-Input Single-Output Gaussian Broadcast Channel (SISO-BC), which is strictly larger than the capacity region achieved by orthogonal transmissions [31]. This increase in the rate is attained through increased complexity in the SIC receivers, since a subset of these have to fully recover messages directed to other users, as illustrated in Figure 2.

### 3.2. Channel Model

The *signal to interference and noise ratio* (SINR) observed by each CU user $C_{i}$ is

(1) $$\Gamma_{i}=\frac{h_{i}p_{i}}{N_{0}+\sum_{k\in D}c_{k,i}h_{k,i}p_{k,i}},\;\forall C_{i}\in C,$$

where $h_{i}$ is the channel gain between user $C_{i}$ and the BS, $h_{k,i}$ is the link gain between the transmitter in MD2D group $D_{k}$ and the BS on channel *i*, $p_{i}$ (respectively, $p_{k,i}$) is the transmission power used for user $C_{i}$ (by the transmitter in $D_{k}$ on channel *i*), $N_{0}$ is the noise density and

(2) $$c_{k,i}=\left\{\begin{array}{cc} b_{k,i}, & \;\mathrm{if}\;\left|h_{k,i}p_{k,i}|<|h_{i}p_{i}\right|,\\ 0, & \;\mathrm{otherwise}, \end{array}\right.$$

where $b_{k,i}=1$ if the MD2D group $D_{k}$ uses channel *i* (and 0 otherwise). Note that power-domain NOMA [6,7] is applied in (2), since the interference caused by stronger interferers—those having $\left|h_{k,i}p_{k,i}|>|h_{i}p_{i}\right|$—is suppressed. Recall that, with SIC, the receiver decodes first the stronger signals, subtracts them from the received signals, and reduces the interference to that caused by the weaker transmitters.

Similarly, the SINR observed in channel *i* for each receiver node $j\in D_{k}$ is given by

(3) $$\Gamma_{k:j,i}=\frac{g_{k:j,i}p_{k,i}}{N_{0}+\tau_{k:j,i}p_{i}\beta_{k:j,i}+\sum_{\ell \neq k}\delta_{\ell ,i}p_{\ell ,i}g_{\ell :j,i}}.$$

In this case, the channel link gains are $g_{k:j,i}$ (the gain between transmitter *k* and receiver $j\in D_{k}$ over channel *i*), and $\beta_{k:j,i}$, the gain between user $C_{i}$ and receiver $j\in D_{k}$. The indicator variables at the denominator are

(4) $$\tau_{k:j,i}=\left\{\begin{array}{cc} 1, & \;\mathrm{if}\;\left|p_{i}\beta_{k:j,i}|<|g_{k:j,i}p_{k,i}\right|,\\ 0, & \;\mathrm{otherwise}; \end{array}\right.$$

and

(5) $$\delta_{\ell ,i}=\left\{\begin{array}{cc} b_{k,i}, & \;\mathrm{if}\;\left|g_{\ell :j,i}p_{\ell ,i}|<|g_{k:j,i}p_{k,i}\right|,\\ 0, & \;\mathrm{otherwise}. \end{array}\right.$$

To simplify notation, we denote the total observed interference as $I_{k:j,i}=\tau_{k:j,i}p_{i}\beta_{k:j,i}+\sum_{\ell \neq k}\delta_{\ell ,i}p_{\ell ,i}g_{\ell :j,i}$, so that $\Gamma_{k:j,i}=\frac{g_{k:j,i}p_{k,i}}{N_{0}+I_{k:j,i}}$.

Note that, in (2), (4) and (5), we assume perfect knowledge of channel state information (CSI). Further, the transmitters follow a distributed NOMA strategy, as shown in Figure 2, since different transmitters (the BS in each of the CUs’ resource blocks or transmitters in other MD2D groups) encode their signals independently but with a common codebook. For the receivers, (2), (4) and (5) imply that those in different groups can use different SIC orderings for decoding, since the channel qualities vary.

### 3.3. Resource Allocation Problem

Our objective in this work is to maximize the energy efficiency of both the MD2D groups and the CUs under the constraint of a minimum rate requirement for every user. This minimum rate may be different for each group or CU, since individual groups or CU users may have diverse data rate requirements depending on the type of their applications.

The (normalized) energy efficiency, measured in bit/Hz/J, is defined for each CU user $C_{m}$ as the ratio of its normalized transmission rate to the energy consumed:

(6) $${\mathrm{EE}}_{C}\left(m\right):=\frac{r_{m}}{u_{m}+p_{m}}=\frac{\log_{2}\left(1+\Gamma_{m}\right)}{u_{m}+p_{m}},\;\forall C_{m}\in C,$$

where $r_{m}$ denotes the transmission rate in bit/s/Hz of user $C_{m}$, $u_{m}$ is the residual power consumed by user $C_{m}$ when there are no data to transmit (a constant) and $p_{m}$ is its transmission power. For the MD2D group $D_{k}$, its energy efficiency is given by

(7) $${\mathrm{EE}}_{D}\left(k\right):=\frac{R_{k}}{v_{k}+\sum_{m\in C}P_{k}^{\left(m\right)}}=\frac{|D_{k}|\sum_{m\in C}\min_{j\in D_{k}}\log_{2}\left(1+\Gamma_{k:j,m}\right)}{v_{k}+\sum_{m\in C}P_{k}^{\left(m\right)}},\;\forall D_{k}\in D,$$

where $v_{k}$ denotes the used power by the transmitter and the receivers in $D_{k}$ group at rest and $P_{k}^{\left(m\right)}$ is the assigned transmission power to the transmitter in group $D_{k}$ over channel $C_{m}$. Note that the rate $R_{k}$ supportable in group *k* (the numerator in (7)) is limited by the receiver with the poorest channel quality, and scales with the size of the group.

The global energy efficiency (GEE) of the cellular network is the ratio between the aggregated rate and the total power needed. Thus, the optimization problem for maximizing the GEE is formulated as

(8) $$\max_{p,\;P_{k},B}\frac{\sum_{m\in C}\log_{2}\left(1+\Gamma_{m}\right)+\sum_{k\in D}\left|D_{k}\right|\sum_{m\in C}\min_{j\in D_{k}}\log_{2}\left(1+\Gamma_{k:j,m}\right)}{\tau \sum_{m\in C}p_{m}+\sum_{k\in D}\sum_{m}b_{k,m}P_{k}^{\left(m\right)}}$$

(9a)subject to bk,m∈{0,1},∑m∈Cbk,m≤s,∑k∈Dbk,m≤r,      k∈D,m∈C(9b)                    pm≤p¯,                                                                      m∈C(9c)                   ∑m∈CPk(m)≤P¯k,                                                    k∈D(9d)                   log2(1+Γm)≥r¯m,                                                  m∈C(9e)                   |Dk|∑m∈Cminj∈Dklog2(1+Γk:j,m)≥R¯k,            k∈D

over the variables $p=(p_{1},\cdots ,p_{M})$ (the power vector allocated to the CUs), $P_{k}=\left(P_{k}^{\left(1\right)},\cdots ,P_{k}^{\left(M\right)}\right),\;k=1,\cdots ,K$, (the power vector allocated to the designated transmitter of group $D_{k}$ over the *M* channels) and $B=[b_{k,m}]$, the K×M channel allocation matrix. The constant $\tau =\sum_{m\in C}u_{m}+\sum_{k\in D}v_{k}$ is just the power consumption of all the devices when there is nothing to transmit. Observe that both CU users and MD2D groups must satisfy individual average power and minimum transmission rate constraints (9b)–(9e).

A second system performance metric is the max-min fair energy efficiency (MMF-EE), which is defined as ${\mathrm{EE}}_{\mathrm{mmf}}=\min\left\{\min_{m}{\mathrm{EE}}_{C}\left(m\right),\min_{k}{\mathrm{EE}}_{D}\left(k\right)\right\}$. Clearly, the MMF-EE uniformly lower bounds the individual EE of all the users, so it provides a common quality of service to all the devices in the network. The corresponding optimization problem is, therefore, to maximize this minimum EE:

(10) $$\max_{p,\;P_{k},B}{\mathrm{EE}}_{\mathrm{mmf}}$$

over the same variables as (8) and constraints (9a)–(9e).

The joint power control and resource allocation is a mixed integer non-linear problem (MINLP) which is NP-hard as proved in [19]. Therefore, we decompose it into two sub-problems: (i) resource allocation; and (ii) power control. In our approach, a (sub-optimal, in general) feasible allocation of channels to MD2D groups is calculated in the first stage; then, in the second or inner stage, the optimal power for each transmitter in the system that maximizes the EE is obtained by successive convexification of the objective function and solving fractional programming problem. This process is iterated until convergence, which is guaranteed and, in our numerical tests, is reached in just a few rounds.

## 4. Resource Allocation: Power Control and Channel Assignment

Our schema to solve the joint power control and channel assignment problem is an alternating optimization between the discrete part and the continuous part. The latter is solved optimally given a fixed channel allocation, and, once the transmission power have been determined, the channels and users (UEs and MD2Ds) are paired. This process is repeated until convergence.

### 4.1. Power Control Algorithm

Suppose a fixed, static channel allocation matrix B. Under this condition, both problems (8) and (10) seem standard convex optimizations, since (9b)–(9e) define a convex solution space, but on closer inspection it can be seen that the objective function is really not concave in p. However, a two-step transformation suffices for converting (8) into a convex problem, and then any classical interior point method is applied to efficiently obtain its solution. Specifically, (8) is first written as $\max_{p\in P}f\left(p\right)/g\left(p\right)$, where g(p) is affine. Should the numerator be concave, the well known Dinkelbach’s algorithm [32] (reproduced here for completeness as Algorithm 1) could readily be used to find the solution, but concavity does not hold in this case as pointed. Thus, at every iteration of Algorithm 1 (Line 3), a concave local approximation $\tilde{f}\left(\;\cdot \;\right)$ is used in place of the true f( · ). Specifically, let $p_{k}\left(B\right)$ be the current approximation at iteration *k* to the optimal vector of transmission powers $p^{*}\left(B\right)$ when the channels are shared as B dictates. Then, f(p) is substituted by a concave $\tilde{f}\left(\;\cdot \;\right)$ such that $\tilde{f}\left(p_{k}\left(B\right)\right)=f\left(p_{k}\left(B\right)\right)$ and a new vector $p_{k+1}\left(B\right)$ of power values is obtained as the maximizer point. We refer to the work in [4,33] for the proof that this procedure converges and finishes in linear time. Note that, for the optimal power vector and multiplier $\left(p^{*},\lambda^{*}\right)$, we have $f\left(p^{*}\right)-\lambda^{*}g\left(p^{*}\right)=0$.

We conclude our discussion of the power control algorithm with the observation that, although the procedure is unchanged with respect to Hmila et al. [4], the outcome of the algorithm is *different* with OMA and NOMA for a fixed channel usage matrix B, because the effective interference appearing in (1)–(3) is lower with NOMA, and the corresponding SINR is higher. The impact of this is discussed below when the numerical results are presented.
**Algorithm 1:** Optimal power control for GEE and MMF-EE (Dinkelbach’s algorithm).1:ϵ>0, λ=0, F=∞2:**while**F>ϵ**do**3:    $p^{☆}=\arg\max_{p\in P}\left\{f\left(p\right)-\lambda g\left(p\right)\right\}$4:    $F=f\left(p^{☆}\right)-\lambda g\left(p^{☆}\right)$5:    $\lambda =f\left(p^{☆}\right)/g\left(p^{☆}\right)$6:**end while**

### 4.2. Centralized Channel Assignment: Matching Theory

We present two complementary methods to calculate the assignment of channels to users. The first resorts to matching theory and is centralized, a single entity (e.g., the BS) is in charge of selecting the best pairs CUs-MD2Ds on a given channel. Matching theory has been previously applied to this setting [34,35], but mostly in the one-to-one and one-to-many variants. In this paper, we also need to consider a many-to-many matching between CUs and MD2D groups in order to capture the *General Case* introduced in Section 2. The simpler resource sharing policies of *Dedicated CUs* and *Distributed Groups* are adequately covered by one-to-one and one-to-many matchings, respectively.

The preference function used to compute the matching is key for the accuracy of the solution. Heuristically, we use for this purpose the aggregate interference level measured at MD2D receivers and CUs. Our rationale is that mitigating the interference entails less power for a given transmission rate. In the interference-limited regime (low or medium SINR), the rate is approximately linear in the SINR level, so we expect that the matching selected on the basis of less accumulated interference is close to optimal. Our numerical simulations confirm this observation (see also [4,19]).

#### 4.2.1. Channel Assignment Algorithm

Formally, a channel assignment is a function between the set of MD2D groups $D_{k}\in D$ and the downlink channels, here identified with the set C. Pairings that cause the minimum possible mutual interference are preferred, according to the following relationships.

**One-to-One Matching:** A one-to-one match μ is a mapping from D∪C to itself such that, for any $D_{k}\in D$, if $\mu \left(D_{k}\right)\neq D_{k}$, then $\mu (D_{k})\in C$, and, if $\mu \left(C_{m}\right)\neq C_{m}$ for some $C_{m}\in C$, then $\mu (C_{m})\in D$. The partner $D_{k}$ is referred to as $\mu (C_{m})$ if $\mu \left(C_{m}\right)=D_{k}$. The preference function for setting the matching uses the received aggregate interference on each MD2D group $D_{k}$ given by
  (11) $$\alpha_{k}^{\left(m\right)}=\max_{j\in D_{k}}I_{k:j,m},\;\;\;\;\forall D_{k}\in D,$$
  for channel *m*. In an analogous form, the aggregated interference seen by each CU user $C_{m}$ is
  (12) $$\Gamma_{m}=\sum_{k\in D}c_{k,m}h_{k,m}p_{k,m},\;\;\;\;\;\forall C_{m}\in C.$$
  Since $\Gamma_{m}$ is additive, we isolate the contribution of the MD2D group $D_{i}$ by denoting $\Gamma_{m}^{D_{i}}=h_{i,m}p_{i,m}$, or, equivalently, setting $c_{i,m}=1$ and $c_{k,m}=0,\;\forall k\neq i$, in (12). Then, the preference relationship is defined as follows: (i) group $D_{k}$ prefers channel $C_{i}$ to $C_{j}$ if $\alpha_{k}^{\left(i\right)}<\alpha_{k}^{\left(j\right)}$; and (ii) user $C_{m}$ prefers group $D_{i}$ to $D_{j}$ if $\Gamma_{m}^{D_{i}}<\Gamma_{m}^{D_{j}}$. Note that, if a channel *m* is empty, then $\mu \left(C_{m}\right)=C_{m}$, and, when group $D_{k}$ is forbidden to transmit on any channel, $\mu \left(D_{k}\right)=D_{k}$.
**Many-to-One Matching:** A many-to-one match μ is a mapping from D∪C to itself such that, for each $D_{k}\in D$, if $\mu \left(D_{k}\right)\neq D_{k}$, then $\mu (D_{k})\in C$, and, if $\mu \left(C_{m}\right)\neq C_{m}$ for some $C_{m}\in C$, then $\mu (C_{m})\in D$. The partner $D_{k}$ is referred to as $\mu (C_{m})$ if $\mu \left(C_{m}\right)=D_{k}$. For many-to-one matches, the preference relationship is similarly defined as follows: (i) transmitter in $D_{k}$ prefers channel $C_{i}$ to channel $C_{j}$ if $\alpha_{k}^{\left(i\right)}<\alpha_{k}^{\left(j\right)}$; and (ii) user $C_{m}$ prefers $D_{i}$ to $D_{j}$ if $\Gamma_{m}^{D_{i}}<\Gamma_{m}^{D_{j}}$; (iii) $\left|\mu (C_{m})\right|$≤r, where *r* is channel *m*’s reuse factor.
**Many-to-Many Matching:** For matches between arbitrary subsets of D and C, the following preference relationship is defined by: (i) $D_{k}$ prefers channel $C_{i}$ to $C_{j}$ if $\alpha_{k}^{\left(i\right)}<\alpha_{k}^{\left(j\right)}$; (ii) user $C_{m}$ prefers $D_{i}$ to $D_{j}$ if $\Gamma_{m}^{D_{i}}<\Gamma_{m}^{D_{j}}$; (iii) $\left|\mu (C_{m})\right|$≤r, where *r* is channel *m*’s reuse factor; and (iv) $\left|\mu (D_{k})\right|$≤s, where *s* is group $D_{k}$’s split factor.

In the centralized approach, a single controller entity first sets a preference list of the transmitters over C and a preference list of the channels/CUs over the *K* groups. If there is no sharing (this corresponds to r=s=1, or dedicated CUs), then the central entity simply applies the Gale–Shapley algorithm to obtain a stable matching between channels and MD2D groups [35]. For the resource sharing cases given when r>1 or s>1, multiple transmitters can share a channel and multiple channels can be used by a transmitter, and a many-to-one matching or many-to-many matching is decided with Algorithm 2. Now, in contrast to the dedicated case, the decision to put MD2D group $D_{k}$ into channel $C_{m}$ depends on the co-channel inter-group interference produced by CU $C_{m}$ and possibly other groups $D_{j},j\neq k$, already using the same RB. Likewise, the acceptance or rejection of a channel $C_{m}$ for a new MD2D group may differ according to the aggregated interference (12). Thus, Algorithm 2 executes the Gale–Shapley exchange iteratively, and in each round one MD2D group is selected and assigned. To that end, the controller first calculates interference as if it would in a one-to-one matching. During the round, those groups whose rank in the channels’ preference lists is not the highest are deferred until some next iteration, and the SINR are recalculated based on the current assignment to evaluate the increase on channel $C_{m}$ due to the addition of some of the unmatched groups. Actually, all the values of the aggregated interference are re-evaluated, irrespective of the status of the group, matched or unmateched. Note that, through the use of (11) and (12), the matching algorithm is considering the SIC decoding for the evaluation of the interference levels. This is an important difference with the previous literature on the use of matching theory for wireless networks [18].
**Algorithm 2:** NOMA interference-based matching algorithm.1:Set up preference list $D2MD_{PL}$, $\forall D_{k}\in D$, from (11)2:Set up preference list $CUE_{PL}$, $\forall C_{m}\in C$, from (12)3:Initial unmatched groups list L=D4:Free groups list F=∅5:r←16:**while**$C_{m}\;\mathrm{occupancy}<r$**do**7:    **while** L≠∅ **do**8:        **repeat**9:           **if** $C_{m}\;\mathrm{occupancy}<r$ **then**10:               Allocate $C_{m}$ to group $D_{k}$;11:           **else if** $C_{m}$ is allocated to $D_{k^{\prime }}$ and $D_{k}$ is preferred over $D_{k^{\prime }}$ **then**12:               Reject $D_{k^{\prime }}$13:               Keep $D_{k}$14:               $L\leftarrow L\cup \{D_{k^{\prime }}\}$15:           **else**16:               Keep $D_{k^{\prime }}$ and reject $D_{k}$17:           **end if**18:           Update $|D_{k}|$ and $|D_{k^{\prime }}|$19:           **if** $|D_{k}|=s$ **then**20:               $L\leftarrow L\setminus \{D_{k}\}$21:           **else if** $\forall C_{i}\in C$, $D_{k}$ is rejected **then**22:               $F\leftarrow F\cup \{D_{k}\}$23:           **end if**24:        **until** all preferences of group $D_{k}$ are tested or $|D_{k}|=s$25:    **end while**26:    Calculate interference values (11) and (12)27:    L=F28:    r←r+129:**end while**

#### 4.2.2. Stability

The matching algorithm produces a stable outcome if no two pairs of agents $\left(c_{1},d_{1}\right)$ and $\left(c_{2},d_{2}\right)$ can be found such that the global utility function increases by swapping the pairings, namely using $\left(c_{1},d_{2}\right)$ and $\left(c_{2},d_{1}\right)$ instead. If some pair with this property exists, it is called a *blocking pair*, so the partnership μ is stable if none pair ($D_{k},C_{m}$) forms a blocking pair for any $D_{k}\in D$ and $C_{m}\in C$ that are not currently matched to each other. This means that both group $D_{k}$ and channel $C_{m}$ prefer each other more than their current partners in the matching. To prove the stability we need to show that these two conditions cannot hold simultaneously. Assume that $D_{k^{\prime }}$ prefers $C_{m}$, so it sends a preference message to $C_{m}$ based on its individual preferences μ( · ). Consequently, $\mu \left(D_{k}\right)\neq C_{m}$ as $D_{k}$ has a lower priority by $C_{m}$ according to μ. This shows that, even though $C_{m}$ is $D_{k}$’s favorite partner, $C_{m}$ does not have incentives toward being matched to $D_{k}$. Thus, the first condition fails. The second condition can be proved along the same argument, and the pair ($D_{k},C_{m}$) cannot be a blocking pair for μ, too. Therefore, the relationship and pairings chosen by the matching algorithm are stable.

### 4.3. Distributed Channel Assignment: Coalition Formation

In Section 4.2, we suppose that a central entity has perfect CSI and uses that for running the matching algorithms. In this section, we show that channel assignment problem can be solved almost optimally using coalitional game-theoretic approach. Consider a coalition game G, which is defined by the triplet (N,v,S):

1. N=C∪D is the set of players, with C and D denoting the sets of CUs and MD2D groups.
2. v is the valuation function that gives the value of a coalition in a game. This is a set function that maps each $S_{i}\subseteq N$ to real non-negative number interpreted as the absolute value of the coalition.
3. $S=\{S_{1},S_{2},\cdots ,S_{n}\}$ is the set of formed coalitions namely the coalition structure. Here, each coalition $S_{i}$ is a subset of N ($S_{i}\subseteq N$, for i=1,⋯,n).

In particular, the definition does not imply that two coalitions are disjoint or that all the agents (CUs and MD2D groups) are part of a coalition. Without loss of generality, every singleton can be regarded as a coalition itself. We further recall some basic notions in coalitional game theory, which are useful below for our discussion. First, agents can have transferable utilities if they are allowed to transfer in a lossless way part of their individual utility to other agents. A transferable utility implies that the coalition structure determines completely the value of the coalition, and this value is totally independent of how the remaining players cooperate. A second important consequence for our purposes is that this class of coalition games have a non-superadditive valuation function. In turn, one can conclude from this that the game will never end up forming a grand coalition containing all the players, because a partition would attain a higher aggregated value. In our case, a grand coalition would lead to a high level of interference which would limit the rates. Hence, using interference for payoffs, utilities and valuations provides the correct signals to promote more efficient solutions.

The proposed coalition game applies repeatedly, in an asynchronous and distributed manner, the following two rules for merging new coalitions or dividing existing ones:

**Merge coalitions** 
Any subset of coalitions $\left\{S_{1},\cdots ,S_{l}\right\}$ may be merged whenever the merged form is preferred by the players. The preference relationship is the same defined in Section 4.2.1 for the centralized solution approach.
**Split coalitions** 
Any coalition ${⋃}_{j=1}^{l}S_{j}$ may be split whenever the split form is preferred by the players.

The principle that lies under the rule for coalition formation is that some MD2D group $D_{k}$ can depart from coalition $S_{i}$ and become a new member of coalition $S_{j}$ only if these conditions are met:

1. The weakest receiver in group $D_{k}$ suffers less individual interference if the group is moved to $S_{j}$ (recall the the receiver with the poorest channel sets the transmission rate for the group).
2. The total mutual interference of coalition $S_{j}\cup D_{k}$ does not increase: $v\left(S_{j}\cup D_{k}\right)\leq v\left(S_{j}\right)$. This condition is essential for guaranteeing convergence of the algorithm. It simply states that movements among coalitions are allowed only if they improve the local value of the coalition.
3. The new coalition structure $S^{\prime }$ results in less total interference than the current one: $v\left(S^{\prime }\right)\leq v\left(S\right)\Rightarrow S^{\prime }$ is preferred to S. In other words, only movements that also improve the total value of the network are approved.

The splitting of some formed coalition follows exactly the same three conditions, but reversing the direction (or the indices).

In Algorithm 3, we initially assume that each coalition has either one CU or one MD2D group with no other members. Then, these individual players take actions i.e, to merge and/or split until *M* coalitions emerge where each coalition has a single CUs and multiple MD2D. According to the constraints (see Algorithm 3), every coalition can host a maximum of *r* MD2D groups, and conversely a maximum of *s* coalitions have a given group as one of its members. In view of the conditions for the formation of cooperative coalitions, their members will likely be a subset of users whose co-channel interference (both intra- and inter-group) is a local minimum. Although minimizing interference is not equivalent to maximizing energy efficiency, two reasons support this choice. Firstly, it is clear that a lower interference level from transmitters in other groups will lead to use potentially less transmission power in a given group sharing the same channel(s) (for a given target rate). Keeping the rate constant (or increasing it) with less consumption of energy gives obviously improved EE. Note that this is basically a greedy argument, since the EE is increased locally, but it is plausible that the global EE also improves in a majority of the network configurations. Secondly, the minimum transmission rates are more easily achievable when the interference level is kept under tight control. This implies that the outcome of the coalition game is less sensitive and more robust to imperfect or delayed CSI. Algorithm 3 always converges to a stable set of coalitions. Here, a stable coalition is defined as one in which the coalition structure cannot be further changed by the merge or split actions presented above, and such that it maximizes the sum of utilities for the players. We refer the readers to the work of Hmila et al. [4] for a proof of this result.

### 4.4. Algorithmic Complexity

The resource allocation sub-problem algorithms, both the semi-distributed and the centralized, terminate after at most *r* rounds, where *r* is the reuse degree. In each round, the semi-distributed algorithm performs a merge and/or split move, while the centralized accepts or rejects a new member. This is repeated for at most *M* channels for all the coalitions or the free groups, so the complexity is no larger than $O(MK^{2})$. The power control sub-problem is solved through the Dinkelbach’s algorithm, which has a sublinear complexity, i.e., superlinear convergence rate [36], and the evaluation of the EE functions takes constant time. The number of variables/constraints is O(M+K) (rate and power for each user). Therefore, the overall worst-case complexity is $O(rMK^{2}\left(M+K\right))$.
**Algorithm 3:** NOMA based merge-and-split for the coalitional game.1:$r_{t}\leftarrow 1$; $S_{m}\leftarrow \left\{\right\}$, m=1,…,M2:**repeat**3:    **while** $r_{t}\leq r$ **do**4:        Identify a feasible $p_{k}^{\left(m\right)}$, $p_{m}$, k=1,…,K, m=1,…,M.5:        Set $R_{k}=\sum_{m\in C}\log_{2}\left(1+\Gamma_{m}\right)+\sum_{k\in D}\left|D_{k}\right|\sum_{m\in C}\min_{j\in D_{k}}\log_{2}\left(1+\Gamma_{k:j,m}\right)$6:        Set $r_{m}=\log\left(1+\Gamma_{m}\right)$, m=1,…,M7:        Sort $S_{m}$ in ascending order using (11)8:        **repeat**9:           **for** m=1,…,M **do**10:               Choose $D_{k}\in S_{m}$11:               **if** $v\left(S_{m}\setminus D_{k}\right)<v\left(S_{m}\setminus D_{k^{\prime }}\right)$ for some $D_{k^{\prime }}$ **then**12:                   $S_{m}\leftarrow S_{m}\setminus D_{k}$13:               **end if**14:               Choose $D_{k}\notin S_{m}$15:               **if** $v\left(S_{m}\cup D_{k}\right)<v\left(S_{m}\cup D_{k^{\prime }}\right)$ for some $D_{k^{\prime }}$ **then**16:                   $S_{m}\leftarrow S_{m}\cup D_{k}$17:               **end if**18:           **end for**19:           Update aggregate interference values with (11) and (12)20:        **until** all $D_{k}\in S_{m}$ are tried **or**$|\{D_{k}:D_{k}\in S_{m}\}|=s$21:        $r_{t}\leftarrow r_{t}+1$22:    **end while**23:**until**$S=\{S_{1},\ldots ,S_{M}\}$ is a stable coalition structure24:Optimize $p_{k}^{\left(m\right)}$, $p_{m}$, k=1,…,K, m=1,…,M, using Algorithm 1

## 5. Numerical Results

In this section, we numerically evaluate the performance of both the centralized and the semi-distributed resource allocation approaches when NOMA is involved using MATLAB and the CVX mathematical optimization package.

We simulated a cell of radius equal to 500 m with λ=250 users (CUs and D2D users). Users are spatially distributed following a standard homogeneous Poisson point process (PPP) [37,38]. Different from other works in the literature, we used two different clustering techniques to form the groups: *K*-Nearest Neighbor (KNN) and Distance Limit (DL) algorithms. Initially, head clusters (the transmitters per group) are randomly selected with both techniques. However, whereas KNN permits to form homogeneous (equal sized) groups, DL defines the group area as a disk around the group transmitter, so it allows us to consider groups of different sizes, including unicast transmissions as a special case. Based on previous results [39], we remark that the clustering technique (i.e., using KNN or DL) usually has little impact on the individual or aggregate behavior [39]. Therefore, we mostly present our results for only one of the algorithms, with similar conclusions being valid for the other one. For resource sharing, CUs having the best channel qualities are selected to share their RBs with MD2D groups. The received signals are assumed to weaken with path loss according to $P_{r}=P_{t}\left(1+{\left(d/d_{0}\right)}^{\alpha }\right)$ where $P_{r}$ is the received power, $P_{t}$ is the transmitted power, $d_{0}$ is a reference distance ( 100 m in our case) and α≥2 is the path loss exponent. The rest of physical system parameters are summarized in Table 2.

### 5.1. Distributed Resource Allocation

**Figure 3:**
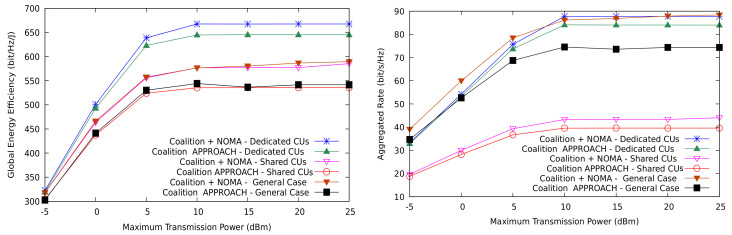
Global energy efficiency and aggregated rate using KNN clustering.

Figure 3 illustrates the impact of NOMA in the semi-distributed (coalition-based) approach for the GEE and the aggregated rate. The spatial configuration used K=4 and K=5 groups, a number of CUs between 2 and 5, and a minimum rate of 0.1 bit/s/Hz. As shown, NOMA improves the GEE within the various resource sharing settings. Similarly, the aggregated rate significantly increases with NOMA (when compared to treating interference as noise), around a 30% in the general sharing case where the reuse of channels implies that receivers can exploit SIC for decoding weak signals. Note that, since the GEE remains almost constant, this means that the higher consumed energy is effectively converted into communication rate, as is expected with NOMA.

**Figure 4:**
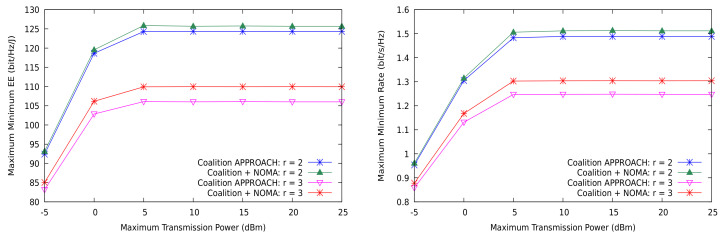
${\mathrm{EE}}_{\mathrm{mmf}}$ and max-min rate for the shared CUs case using DL clustering.

The minimum rate and ${\mathrm{EE}}_{\mathrm{mmf}}$ are depicted in Figure 4 for the coalition-based approach when NOMA is used in a shared CUs setting with r=2 and r=3. This time, the clusters are formed using DL, with the average number of receivers set to 3. NOMA helps to increase ${\mathrm{EE}}_{\mathrm{mmf}}$ and the transmission rate simultaneously, but the gain is clearly larger if the reuse factor *r* grows, since this allows for more degrees of freedom when sharing the channels. Therefore, NOMA can only realize its full potential when the co-channel interference level prevents the system to attain the target transmission rates, while it just provides a marginal gain in the high SNR regime. As a consequence, NOMA is mostly useful in dense wireless systems when the number of active users is high, even if the transmission rate required by the devices is not particularly high (e.g., mMTC in 5G).

**Figure 5:**
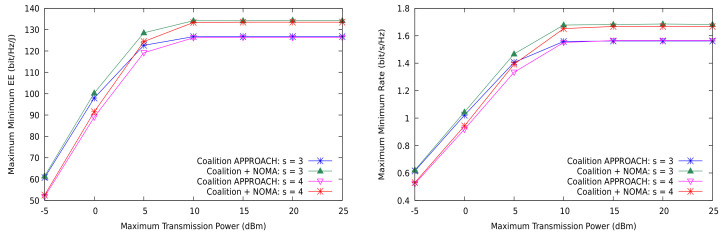
${\mathrm{EE}}_{\mathrm{mmf}}$ and max-min rate for the general case using DL clustering.

**Figure 6:**
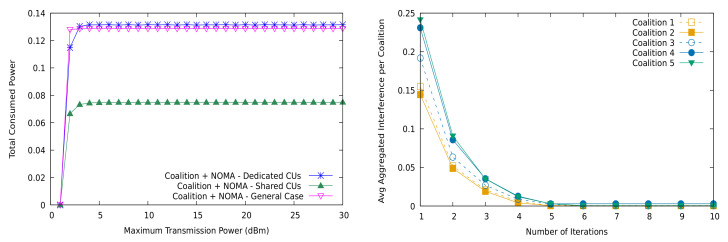
Total consumed power and interference level per coalition with NOMA and the distributed coalition game.

For the general sharing scenario (r=2, s=3,4), Figure 5 shows that using NOMA is not detrimental to the ${\mathrm{EE}}_{\mathrm{mmf}}$ or the worst rate for any receiver. We used 6–8 CUs and 4 MD2D groups in both simulation cases. Further, note that the minimum rate per channel was set to 0.5bit/s/Hz. Thus, although using NOMA leads the network to consume more power for transmitting, this is again not wasted; it is instead used to achieve the spatial degrees of freedom of the multi-user channels. Figure 6 shows precisely the increase in the consumed power between the partial sharing (shared CUs) and the full sharing (general) cases, confirming that aggregate interference is not only well controlled by the allocation algorithm (right), but also used to extract information under receiver SIC.

**Figure 7:**
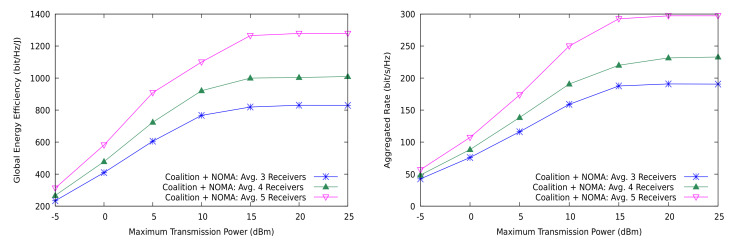
Global energy efficiency and aggregated rate with NOMA and the distributed coalition game for the general case with different group sizes.

The impact of NOMA is also positive as the group sizes increase in the general sharing scenario (Figure 7). Both the GEE and the achieved sum-rate improve substantially with more receivers per group, and the increase is faster in the low SNR region. We emphasize that the results are shown for an *average* number of receivers per group, since the device locations and the cluster formation are random. With the parameters listed in Table 2, the maximum distance between the receiver and the transmitter happens to be within 50–80 m.

### 5.2. Comparison to Optimal Resource Allocation

We now analyze the performance of NOMA compared to the optimal centralized resource allocation solution. Although the centralized approach is not generally applicable in large networks, its performance results set a baseline to assess the achievable gains with NOMA and SIC with respect to the system parameters (i.e., the number of groups and receivers per group).

**Figure 8:**
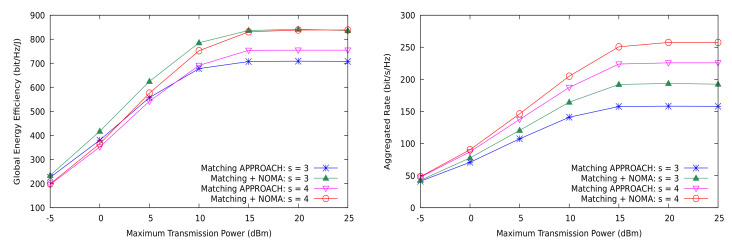
Global energy efficiency and aggregated rate with the matching theory based solution vs. NOMA for the general case.

**Figure 9:**
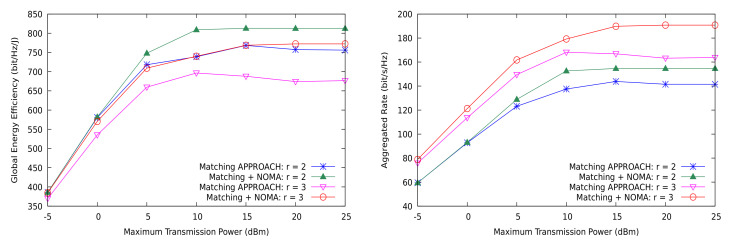
Global energy efficiency and aggregated rate with the matching theory based solution vs. NOMA for the shared CUs case.

First, we show in Figure 8 that NOMA is able to simultaneously attain better global energy efficiency and higher sum-rate than OMA in the network. This implies that NOMA better approaches the capacity region of the MD2D cell with similar or lower consumption of energy, thus demonstrating that this communication strategy clearly outperforms OMA in this scenario. However, the optimality of NOMA is still unclear since we do not currently know the exact capacity region of the system for arbitrary sizes. Accordingly, the plots in Figure 8 must be considered as an achievable inner approximation, yet with a gap to the theoretically optimal system performance. We discuss below whether NOMA alone can reasonably be the best encoding and decoding strategy for optimizing multi-user communication systems. The gain over OMA is also notable in the case of shared CUs, or partial sharing, in both of the metrics (see Figure 9). In addition, observe that the difference between NOMA and OMA (referred to as the matching approach) increases with *r*, the reuse factor.

**Figure 10:**
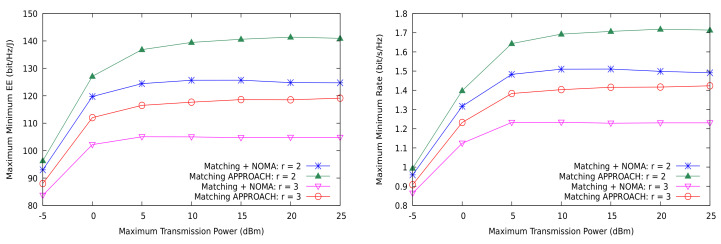
${\mathrm{EE}}_{\mathrm{mmf}}$ and max-min rate with the proposed matching theory based solution vs. NOMA for the shared CUs case.

Nevertheless, for the max-min performance metrics, the conclusions are slightly different. As shown in Figure 10, ${\mathrm{EE}}_{\mathrm{mmf}}$ is actually worse with NOMA than with OMA for a given number of channel sharing degrees of freedom (only the cases of r=2 and r=3 are shown). We recall that NOMA can use more power in the transmitters, since part of the interference is removed by SIC processing at the receivers, but, even under perfect SIC and strict power control, the receivers with stronger channels can experience lower $E_{\mathrm{mmf}}$. This is not as unfavorable as it appears, because Figure 10 (right) shows that the max-min rate actually increases with NOMA.

## 6. Discussion

The results presented in the previous section show that, when channel reuse is allowed in MD2D communications, NOMA and SIC-enabled receivers can attain higher sum-rate than OMA in a consistent manner, i.e., almost independently of the number of groups and the number of receivers per group. This is possible at the same energy efficiency for the network, but not generally for the MMF energy efficiency, which can be worse in some cases. However, the max-min rate is still improved with NOMA, although at the cost of consuming more energy.

In modern wireless communication systems, transmitters and receivers come equipped with multiple antennas. Our model only considers single-antenna sources and receivers, so it is natural to investigate the role of NOMA and MIMO together, since it is well known that NOMA is optimal from an information theoretical point for the single-user broadcast channel. Given that MIMO and NOMA are far better than point-to-point and OMA systems, respectively, it seems plausible that their combination yields even better benefits, at least in the sum multiplexing rate. Contrary to intuition, some recent works [40,41] unveil several shortcomings and misconceptions about multi-antenna NOMA and show that linear precoding (for the downlink) and/or rate-splitting can be strictly better than NOMA in many common network settings. Moreover, both rate splitting and precoding require less complex receivers, so their implementation is easier. In view of this, more research on the contexts where NOMA is preferable over the latter strategies is necessary to determine under which conditions a receiver-only SIC approach is optimal in terms of sum-rate and multiplexing gains. In the special case of MD2D communications, one could consider an adaptive hybrid system where NOMA and rate-splitting are used depending on the number of receivers in the D2D group, for instance, due to the similar complexity of both techniques for a small number of receivers.

## 7. Conclusions

In this paper, we consider the inclusion of NOMA into an optimization framework for designing multicast D2D communication systems. We show first that the base mathematical framework can be adapted without much difficulty to the non-orthogonal multiple access case for the downlink part, and that the centralized and distributed optimization procedures only need some technical changes to work properly. Next, we conducted a set of numerical experiments to evaluate the performance gains achievable with NOMA in our context, focused on global and max-min energy efficiency, and global and max-min sum rate for the receivers. Our results suggest that NOMA is efficient for the sum rate (in both cases) and for the global energy efficiency, but in contrast it may not be efficient for the max-min energy efficiency. Moreover, our results were obtained under QoS constraints for the rate, thus MD2D with NOMA can cope with high system-level throughput, reliability and heterogeneity in 5G wireless networks. While our focus was on the downlink communication, the same model and techniques can be used for the uplink direction, where the BS applies SIC for separating the signals. Nevertheless, this is a single-receiver model which has already received much attention in the literature.

While the results in the paper highlight the performance improvements that NOMA can offer for reusing the transmission channels among disjoint groups of co-located (or nearby) transmitters–receivers, there are still many issues worth investigating before fully understanding the interplay between NOMA and D2D, such as the role (architectural and related to performance) of NOMA in enhanced mMTC and eMBB services, massive IoT with short-packet communications and D2D networks. Performance analysis of NOMA in D2D and MD2D with full-duplex nodes is also a promising research direction.

## Figures and Tables

**Table 1:** D2D pair/groups, MD2D and NOMA: State of the art.

Ref.	Scenario	Approach	Model	Problem	Objective	NOMA
[17]	Dedicated CUs	Optimization	D2D pair	PC,RA	D2D sum rate	CU
[22]	Distributed Groups	Matching Theory	D2D group	PC,RA	Energy consumption, delay	CU
[25]	Distributed Groups	Optimization	D2D pair	RA,PC	Min transmission power	D2D + CUs
[26]	Dedicated CUs	Graph Theory	D2D group	PC,RA	D2D EE	D2D
[24]	Distributed Groups	Match Theory	D2D group	RA	Network sum rate	D2D + CUs
[28]	Dedicated CUs	Optimization	D2D pair	RA +	System sum rate	CU
				Mode Selection		
[29]	Dedicated CUs	Matching Theory	D2D group	RA	System sum rate	D2D
[30]	Dedicated CUs	Game Theory	D2D group	RA	CUs throughput	D2D
[18]	Dedicated CUs	Matching Theory	D2D group	PC,RA	Maximum users SINR	D2D
[27]	Dedicated CUs	Hungarian Algorithm	D2D pairs	PC,RA	D2D energy	D2D

**Table 2:** Simulation parameters.

Parameter	Value
Cell radius	500m
Reuse factor (*r*)	{2,3}
Network density	250 devices/cell
Split factor (*s*)	{3,4}
Path loss exponent	2.5
Minimum transmission rate	{0.1,0.5} bit/s/Hz
Number of CU users (*M*)	{5,6,8}
Maximum transmission powers	[−5,25] dBm
Number of MD2D groups (*K*)	{4,10,15}
Number of receivers $D_{j}$	{3,4,5}
Circuit power	10dBm

## Data Availability

Not applicable.
